# Operative Dentistry

**Published:** 1881-12

**Authors:** J. Hooper


### OPERATIVE DENTISTRY.

BY J. HOOPER.

*Read before the Kentucky State Dental Society, June,* 1881.

Operative Dentistry is a subject of which a great deal has been said and written by the ablest men in the profession, but it is almost exclusively in regard to filling teeth; and, indeed, it seems to be the general impression with the profession that filling teeth covers the whole of operative dentistry. You may look over any of the dental journals, or the transactions of the societies, or any paper or discussion titled, Operative Dentistry, and they are almost invariably about filling teeth; or, you may ask one dentist of another's qualifications as an operative dentist, and he will either say he is, or is not, a fine operator. If he says he is a fine operator and you ask him how he knows, the answer is sure to be, "I saw some of his fillings," or, I saw him filling teeth, and, from that alone he judges of his qualifications as an operative dentist.

Before we go farther we will see what the definition of Operative Dentistry is.

I suppose Harris is accepted as authority.

First, we will see what Operative means, and then, Operative Dentistry. Operative means, "Active, efficacious, practical." (Harris.)

Operative Dentistry means "That department of dentistry which pertains to the surgical treatment of diseases of the teeth and surrounding parts." (Harris.)

Some dentists, I believe, instruct their students that filling teeth covers the whole of operative dentistry; and, judging from the conversation of some of the graduates of the different colleges, you would think this was also taught.

The first thing that requires the attention of an operative dentist is the eruption of the teeth, and this is something that has been neglected more than any part of dentistry. First, an operative dentist should know when the gums need lancing, and when to extract the deciduous teeth to make room for the permanent set when Nature fails to do her duty, and when to fill a deciduous tooth and what with, also, how to correct irregularities without losing any of the natural organs.

The second thing that comes under operative dentistry is cleaning the teeth. It is very important to thoroughly clean the teeth; not only for the health of the teeth but also for the health of the patient, and especially before attempting to fill.

3rd. Next comes filling the teeth, about which so much has been said and written. When we young men start out to practice our profession, we find harmony until we come to this part of operative dentistry, then we find discord. As we try to advance we will read, or hear from one man, that soft foil is the only material for a permanent filling. Another will say that hard foil is the only thing suitable for a permanent filling, and then comes another who says that plastic fillings are the only permanent fillings, and each of these men are, or have been, teachers in our colleges. I think each of these is good for certain classes of teeth, but we all know that there is a great difference in the constitution of the teeth in different individuals, and why not treat each case accordingly?

Last, comes the extraction of the teeth, and this part of operative dentistry is very much abused. I don't think a tooth should be extracted until we have exhausted every means in our power to save it. And, another practice that is very wrong, is the extraction of the six year molars, as I have heard some of the leading men of the profession advocate, especially if the teeth are crowded and have begun to decay; and why not expand the arch and fill the teeth and retain one of the best teeth nature has placed in the mouth.

It is the duty of the operator, when he has to extract a tooth to know why he has to extract it; and, if he cannot tell before he takes it out, after he extracts it break it open and see if he can tell then. I have had a case here that I had to extract and I could not tell why until I broke it open, and that explained the whole matter. The pulp was completely ossified.

I hope these few remarks will induce the members of this Society to give their views on this subject.

J. Hooper.

